# 2301. Viral Kinetics of Sequential SARS-CoV-2 Infections

**DOI:** 10.1093/ofid/ofad500.1923

**Published:** 2023-11-27

**Authors:** Stephen Kissler, James Hay, Joseph Fauver, Christina Mack, Caroline Tai, Deverick Anderson, David D Ho, Nathan Grubaugh, Yonatan H Grad

**Affiliations:** University of Colorado Boulder, Boston, Massachusetts; University of Oxford, Oxford, England, United Kingdom; University of Nebraska Medical Center, Omaha, Nebraska; IQVIA, Durham, North Carolina; IQVIA, Durham, North Carolina; Duke Center for Antimicrobial Stewardship and Infection Prevention, Durham, North Carolina; Columbia University Vagelos College of Physicians and Surgeons , New York, NY; Yale School of Medicine, New Haven, Connecticut; Harvard Chan School of Public Health, Boston, Massachusetts

## Abstract

**Background:**

An estimated 65% of the US population had at least two SARS-CoV-2 infections by November 2022, but the impact of prior infection on disease course in subsequent infections has been debated. Population-level studies can yield conflicting results due to biases that arise from differences in differences in infection history, vaccination status, and patient characteristics. Examining the dynamics of SARS-CoV-2 infections at the individual level can help adjust for these biases.

**Methods:**

Using a convenience sample of 94,812 longitudinal RT-qPCR measurements from samples each taken from a combined anterior nares/oropharyngeal swab, we compared the SARS-CoV-2 viral kinetics of first vs. second infections, adjusting for viral variant, vaccination status, and age. We characterized the kinetics of these infections by fitting a hierarchical Bayesian piecewise linear model to the viral concentration measurements.

**Results:**

Relative to first infections, second infections usually featured a faster clearance time (5.1 days, 95% CI (4.7, 5.7) *vs.* 8.8 days (7.8, 9.8) in first infections), especially in individuals who received a vaccine dose between their first and second infection. Furthermore, a person’s relative (rank-order) viral clearance time, compared to others infected with the same variant, was similar across first and second infections (Spearman correlation: 0.475, *p* < 0.0001); that is, individuals who had a relatively fast clearance time in their first infection tended to also have a relatively fast clearance time in their second infection.

**Conclusion:**

Like vaccination, immunity from a prior SARS-CoV-2 infection shortens the duration of subsequent acute SARS-CoV-2 infections principally by reducing viral clearance time. Additionally, there appears to be an inherent element of the immune response, or some other host factor, that shapes a person’s relative ability to clear SARS-CoV-2 infection that persists across sequential infections.

**Disclosures:**

**Stephen Kissler, PhD**, ModernaTx: Advisor/Consultant **Christina Mack, PhD**, IQVIA: Full time employee of IQVIA which is in paid contractual relationship with bio pharma companies. **Caroline Tai, PhD**, Evidation Health: Stocks/Bonds|IQVIA: Employee **Yonatan H. Grad, MD, PhD**, Day Zero Diagnostics: Board Member|GSK: Advisor/Consultant

